# Total intravenous anesthesia for geriatric hip fracture with severe systemic disease

**DOI:** 10.1007/s00068-023-02291-z

**Published:** 2023-06-24

**Authors:** Yu-Yi Huang, Chung-Kun Hui, Ngi-Chiong Lau, Yuet-Tong Ng, Tung-Yi Lin, Chien-Hao Chen, Ying-Chih Wang, Hao-Che Tang, Dave Wei-Chih Chen, Chia-Wei Chang

**Affiliations:** 1 Department of Orthopedic Surgery, Chang Gung Memorial Hospital, Keelung Branch, No. 222, Maijin Rd., Anle Dist., Keelung City, 204 Taiwan; 2 College of Medicine, Chang Gung University, No. 259, Wunhua 1st Rd., Guishan Dist., Taoyuan City, 333 Taiwan; 3 Department of Anesthesiology, Chang Gung Memorial Hospital, No. 222, Maijin Rd., Anle Dist., Keelung City, 204 Taiwan

**Keywords:** TIVA, Nerve block, Hip fracture, Geriatric

## Abstract

**Purpose:**

Our study aimed to determine the impact of a novel technique of anesthesia administration on the clinical outcomes and complications in geriatric patients with severe systemic disease undergoing hip surgery.

**Methods:**

We retrospectively identified patients aged > 65 years with severe systemic disease that was a constant of life [American Society of Anesthesiologists (ASA) IV] who underwent surgery for hip fracture between January 2018 and January 2020. The patients were divided into two groups: Group I [fascia iliaca compartment block plus propofol-based total intravenous anesthesia (FICB + TIVA)] and Group II [general anesthesia (GA)]. The primary outcomes were 30-day and 1-year mortality. The secondary outcomes included length of hospital stay, length of intensive care unit (ICU) stay, postoperative morbidity, Visual Analog Scale score, and consumption of analgesics.

**Results:**

There was no significant difference in the 30-day mortality (5 vs. 3.8%, *p* = 0.85) and 1-year mortality (15 vs. 12%, *p* = 0.73) between the groups. Group I had significantly lower ICU requirements (*p* = 0.01) and shorter lengths of ICU stay (*p* < 0.001) and hospital stay (*p* < 0.001). Moreover, a smaller proportion of patients in Group I required postoperative morphine or oral opiates.

**Conclusion:**

Geriatric patients who underwent hip surgery under FICB + TIVA required fewer ICU admissions, shorter lengths of ICU and hospital stay, and had lesser postoperative opioid consumption than those who were under GA. Hence, we recommend the novel FICB + TIVA technique for hip fracture surgery in geriatric patients with poor general health status and high surgical risks (ASA IV).

## Introduction

Hip fracture is a common fragility injury in the elderly that is associated with significant mortality and morbidity. Approximately 1.6 million cases of hip fracture occur annually worldwide [1, 2]. The 1-month mortality following a hip fracture is reported to be 4–12% and is as high as 35% at 1 year [3]. With an increase in the aging population, the number of hip fractures will continue to increase. More research is necessary to improve clinical outcomes following surgery for hip fractures.

Management of hip fractures in the elderly is challenging, especially in patients with severe systemic diseases [American Society of Anesthesiologists (ASA) grade III or IV]. The ASA classification is a measure of intraoperative and postoperative risks based on the severity of medical comorbidities. Previous studies have indicated that the ASA score predicts mortality and morbidity in elderly patients who sustain a hip fracture, and patients with a high ASA class (III or IV) have an increased risk of mortality and complications after hip fracture surgery [4–6]. If the hip fracture is left untreated due to medical reasons, the 1-year mortality in such patients could be more than 60% [7–9]. Recent studies have demonstrated that different types of anesthesia, such as spinal anesthesia (SA) and regional anesthesia (RA), can reduce the intraoperative and postoperative complications associated with fragility hip fracture surgery in the elderly compared with those treated under general anesthesia (GA) [4, 5, 10–13]. Previous studies also showed that total intravenous anesthesia (TIVA) has several advantages in terms of recovery in elderly with fragile hip fracture [8, 14, 15]. Patients undergoing surgery under TIVA can be managed using short-acting drugs and supraglottic airway device. The hemodynamic stability is easily maintained and minimizes the risks of postoperative cardiovascular complications and cerebrovascular accidents.

Another concern associated with the surgical management of geriatric hip fractures is the morbidity caused by postoperative opioid consumption. It leads to sedation and reduces the ability of physical therapy. It also has adverse effects, such as delirium, nausea, and vomiting, leading to aspiration pneumonia and the need for critical care. Compared with standard opioid analgesia, peripheral nerve block reduces the use of opioids, improves the Visual Analog Scale (VAS) score, and reduces the length of hospital stay. In patients with a high ASA class (III or IV), peripheral nerve block can decrease the risk of mortality and the need for critical care after hip surgery [16, 17].

According to the American Academy of Orthopedic Surgeons there is no standard type of anesthesia to be used in surgeries for hip fractures. The choice of anesthesia is based on the patient’s comorbidities and physician’s preference [5]. Moreover, there are no recent studies on the influence of anesthesia for hip fracture surgery in the elderly with severe systemic disease (ASA IV). The goal of our study was to assess the clinical outcomes and complications in patients with ASA grade IV undergoing surgery for hip fracture under two different techniques of anesthesia at our hospital.

## Materials and methods

### Study design

The retrospective cohort study was approved by our hospital’s institutional review board (No. 20210799B0). The inclusion criteria included patients aged > 65 years with severe systemic disease (ASA IV) who underwent surgery for low-energy hip fracture (femoral neck, intertrochanteric or subtrochanteric femur fractures) between January 2018 and January 2020. These patients received either fascia iliaca compartment block plus propofol-based TIVA (FICB + TIVA) (Group I, *n* = 20) or GA (Group II, *n* = 26) according to the anesthesiologist’s preference. If patients older than 65 years with severe medical comorbidities, including cardiovascular problems, chronic obstructive lung disease, cardiovascular accident, FICB + TIVA was indicated. We excluded patients with pathologic fractures, polytrauma, prior surgery at the affected hip, bilateral hip fracture, or missing information about the anesthesia type.

Standard monitoring, including pulse oximetry, non-invasive blood pressure monitoring, and electrocardiography, were performed for all patients after arrival to the operative room.

**Fig. 1:**
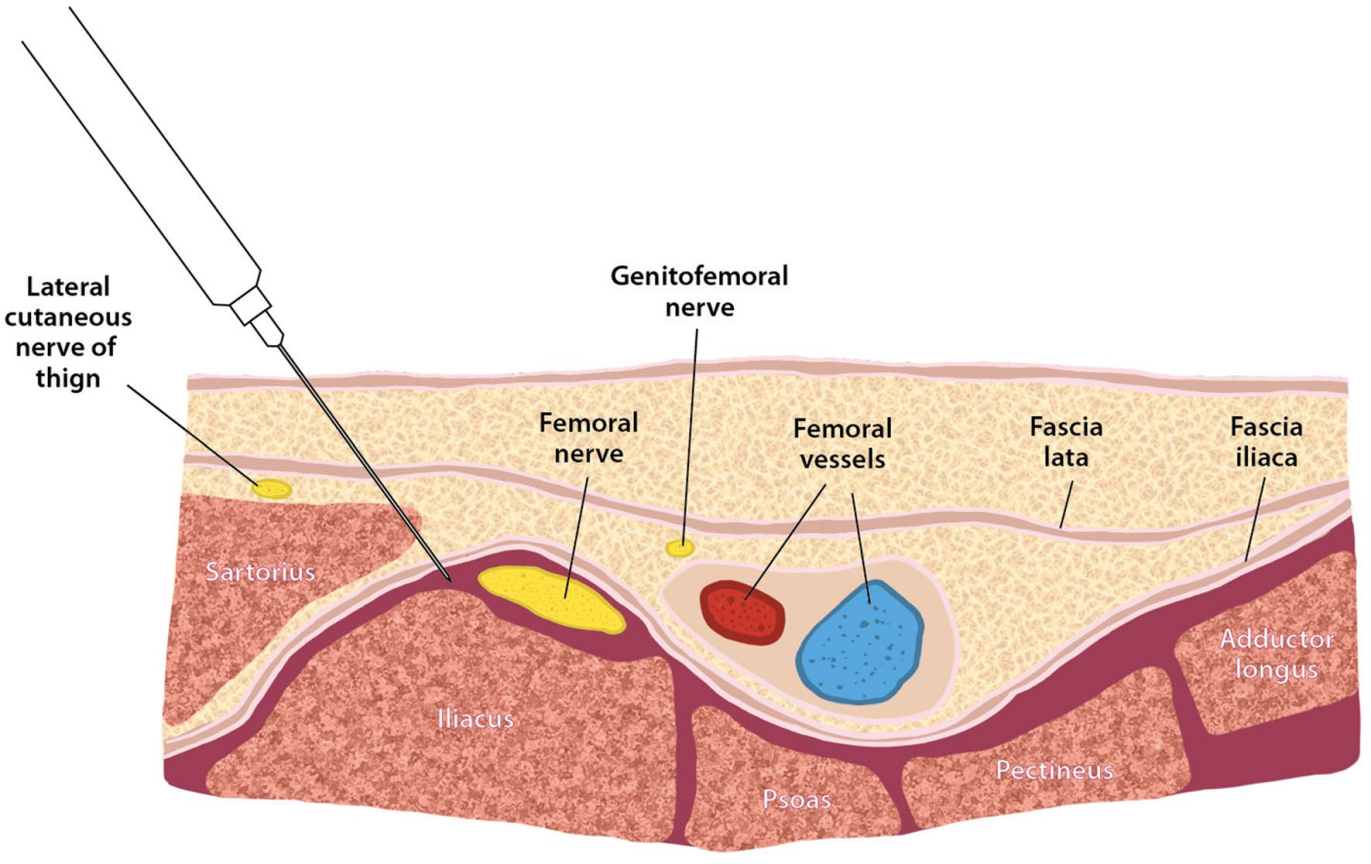
Anatomy of a fascia iliaca compartment block

In Group I, all FICBs were performed preoperatively in the preoperative preparation room under ultrasound guidance at least 30 min before surgery (Fig. 1). The fascia iliaca nerve block was administered as a 25 ml local anesthetic mixture consisting of lidocaine 200 mg, levobupivacaine 50 mg, and normal saline. Subsequently, the patients received propofol, and anesthesia was maintained through a target-controlled infusion throughout the procedure. Oxygen infusion at the rate of 5 l/min was administered via a face mask instead of inserting an endotracheal tube during the procedure. A bolus dose of intravenous 1–2 ml fentanyl (0.05 mg/ml) was used intraoperatively, as tolerated.

In Group II, the patients received intravenous propofol 2 mg/kg, lidocaine 0.4 mg/kg, fentanyl 2 µg/kg, and cisatracurium 0.2 mg/kg for induction, and an endotracheal tube was inserted. The patients were under continuous inhalation of sevoflurane or desflurane for maintenance of anesthesia. At the end of the surgery, the patients were under neostigmine 2.5 mg and atropine 1 mg before extubation of the endotracheal tube. Periarticular blocks or additional local anesthetics were not used during surgery.

### Outcomes

The primary outcomes were 30-day and 1-year mortality. The secondary outcomes included length of hospital stay, need for intensive care unit (ICU) admission, length of ICU stay, postoperative morbidity, postoperative VAS score, and consumption of analgesics. The length of hospital stay was defined as the number of days from admission to discharge, and the length of ICU stays was defined as admission to ICU for critical care to ordinary ward transfer.

### Covariates

The patient covariates included age, sex, hip fracture type, procedure, preoperative waiting time, and comorbidities as identified from our medical records.

### Statistical analysis

The normal distribution of the data was evaluated using the Kolmogorov–Smirnov test, and the independent *t* test and chi-square test were performed for comparing numerical variables and categorical variables, respectively. The results were considered statistically significant if the *p* value was < 0.05. All statistical calculations were conducted using IBM SPSS Statistics 25 (IBM; Armonk, New York, USA).

## Results

There were 46 patients with ASA grade IV enrolled in this study. Among them, 20 patients in Group I received FICB + TIVA, and 26 in Group II received GA. The mean age was 83.8 ± 9.1 years in Group I and 85.6 ± 7.8 years in Group II (*p* = 0.46). Nine patients in Group I and 13 in Group II were female. Most patients had a femoral neck fracture or intertrochanteric fracture and underwent an internal fixation procedure. Time from arrival in the emergency room (ER) to surgery was 79.0 ± 81.4 h in Group I and 76.2 ± 68.2 h in Group II (*p* = 0.41). (Table 1). No patient under FICB + TIVA converted to GA during operation.

**Table 1:** Patient demographic data

	FICB + TIVA Group I (*n* = 20)	GA Group II (*n* = 26)	*p* value
Age (years)	83.8 ± 9.1 (range 70–95)	85.6 ± 7.8 (range 69–97)	0.46
Sex (M/F)	11/9	13/13	0.77
Fracture type			0.29
Femoral neck fracture	10	12	
Intertrochanteric fracture	10	11	
Subtrochanteric fracture	0	3	
Procedure			0.88
Internal fixation	12	15	
Hemiarthroplasty	8	11	
Time from ER to surgery (hours)	79.0 ± 81.4	76.2 ± 68.2	0.41
Comorbidities			
Cancer	3	7	0.34
Cerebral vascular accident	2	7	0.16
Chronic kidney disease	5	10	0.34
Chronic obstructive pulmonary disease	3	4	0.97
Congestive heart failure	3	10	0.08
Coronary artery disease	6	13	0.23
Dementia	6	2	0.06
Diabetes mellitus	8	13	0.50
End-stage renal disease	0	1	0.25
Gouty arthritis	1	1	0.85
Hypertension	14	22	0.24
Liver disease	3	1	0.19
Parkinsonism	0	1	0.38
Peptic ulcer disease	5	4	0.42

*TIVA* total intravenous anesthesia, *FICB* fascia iliaca compartment block, *GA* general anesthesia, *M* male, *F* female, *ER* emergency room

Patients in both groups had significantly lower VAS score after surgery (7.4 ± 0.5 to 2.9 ± 1.0, *p* = 0.003 in Group I and 7.5 ± 0.5 to 3.1 ± 1.2, *p* < 0.001 in Group II). There was no significant difference in VAS score on POD1 (2.85 ± 1.04 vs. 3.12 ± 1.24, *p* = 0.43), POD2 (2.2 ± 0.70 vs. 2.5 ± 0.71, *p* = 0.96), and POD3 (2.1 ± 1.62 vs. 2.73 ± 1.28, *p* = 0.27) in both groups. Group II consumed more amount of postoperative morphine (*p* < 0.001) or oral opiates (*p* = 0.018) for pain control than Group I. Compared with Group I, a higher proportion of patients in Group II had postoperative anemia, which required blood transfusion (*p* = 0.017) and postoperative pneumonia, which required antibiotic treatment and respiratory therapy (*p* = 0.03).

Group I had a shorter hospital stay before discharge to home or healthcare facility than Group II (8.8 ± 3.8 vs. 14.5 ± 17.8 days, *p* < 0.001). Fewer patients (10%) in Group I required ICU admission after surgery than those in Group II (42%) (p = 0.01). Among the patients needed postoperative ICU admission, two patients in Group I were under critical conditions with refractory hypotension. Both of them did not need intubation or a mechanical ventilator during ICU admission. In Group II, patients who fail to extubate immediately (*n* = 6) and post-operative refractory hypotension (*n* = 5) needed ICU care. Two of them need to re-intubate and prolong mechanical ventilator supply due to progressive pneumonia course. Patients in Group I had a shorter ICU stay than those in Group II (2.0 ± 1.4 vs. 7.2 ± 15 days); however, the difference was not significant (*p* = 0.15). No patient in either group died during the hospital stay. Moreover, 63% patients were discharged to home and 37% were discharged to a health care facility. There was no significant between-group difference in 30-day mortality (*p* = 0.85) and 1-year mortality (*p* = 0.73) (Table 2).

**Table 2:** Clinical outcomes and complications

	FICB + TIVA Group I (*n* = 20)	GA Group II (*n* = 26)	*p* value
VAS			
Pre-op	7.4 ± 0.5	7.5 ± 0.5	0.96
POD 1	2.9 ± 1.0	3.1 ± 1.2	0.43
POD 2	2.2 ± 0.7	2.5 ± 0.7	0.96
POD 3	2.1 ± 1.6	2.7 ± 1.3	0.27
*p* value	**0.003**	**< 0.001**	
Use of analgesic agent			
Morphine	3	22	**< 0.001**
Parecoxib	10	10	0.44
Oral opiates agents	16	26	**0.02**
Complications			
Acute myocardial infarction	0	3	0.12
Acute kidney injury	1	4	0.27
Delirium	3	2	0.44
Perforated peptic ulcer	1	1	0.85
Pneumonia	2	10	**0.03**
Postoperative anemia	2	11	**0.02**
Surgical site infection	0	1	0.38
Seizure	0	0	–
Urinary tract infection	2	3	0.87
Hospital stays (days)	8.8 ± 3.8 (range 4–30)	14.5 ± 17.8 (range 5–95)	**< 0.001**
ICU requirement	2	11	**0.01 **
Refractory hypotension	2	5	
Unsuccessful extubation	0	6	
ICU stays (days)	2.0 ± 1.4 (range 1–3)	7.2 ± 15 (range 1–52)	0.15
Discharge deposition			0.33
Home	11	18	
Health care facility	9	8	
30-day mortality	1	1	0.85
1-year mortality	3	3	0.73

*TIVA* total intravenous anesthesia, *FICB* fascia iliaca compartment block, *GA* general anesthesia, *VAS* visual analog scale, *ICU* intensive care unit

*p* values in bold signify a statistically significant difference

## Discussion

With an increase in the elderly population, the incidence of hip fractures continues to increase. The risk factors associated with mortality following a hip fracture include ASA physical status, age, and delay in surgery [4]. Among the elderly population with high ASA status who suffer from hip fracture, delay in surgery might be inevitable because of multiple pre-existing comorbidities. In our study, the duration from ER to surgery was longer than that reported in previous studies (79.0 ± 81.4 h in Group I and 76.2 ± 68.2 h in Group II). This is because most patients with ASA grade IV status had multiple comorbidities and required treatment for their underlying diseases, such as progressive chronic renal disease, stroke, diabetes mellitus with poor control, pneumonia, or gastrointestinal bleeding. It was considered that the low-energy hip fracture was induced by their chronic medical condition [18]. The hip fracture surgery was performed after achieving a relatively stable general health status. It was important to focus on the general health status before surgery to decrease mortality rate. RA or TIVA was potential technique to send patients earlier to the operation room in a previous study [4, 5].

In a previous study, SA seemed more common for the elderly population with hip fracture surgery [8]. However, for the elderly with ASA IV, it was difficult to control the blood pressure during operation and the whole surgical procedure was not smooth under SA. We considered that it was not a reliable option for the patients with ASA IV under SA. Since a certain rate of delirium during hip fracture surgery was another issue in the elderly population under SA, further studies may be designed to compare the rate of delirium between FICB + TIVA and SA.

Many studies compared RA and GA for hip fracture surgery; however, they did not conclude that RA decreased mortality [4, 8, 11]. However, RA was recommended because of the advantages in postoperative outcomes. We compared geriatric patients with ASA grade IV undergoing surgery for hip fracture under TIVA or GA. Lesser need for ICU admission and shorter lengths of ICU stay and hospital stay were observed in Group I. However, there was no significant difference in the 30-day and 1-year mortality rates between both groups. These findings were similar to those of a previous study [4, 5, 8]. Neuman et al. [11] retrospectively reviewed the data of patients aged over 50 years who underwent hip fracture surgery. After matching for the observed patient and hospital factors, RA was not associated with lower 30-day mortality but was associated with a shorter length of hospital stay than GA. Shin et al. [8] randomly assigned patients over 65 years undergoing hip fracture surgery to GA, SA, or TIVA groups. In-hospital, 30-day, and 90-day mortality was not significantly different between the groups. Qiu et al. [5] compared GA and RA for hip fracture surgery in the elderly. Time to death, in-hospital mortality, length of hospital stay, and discharge were all increased by GA. Klavas et al. [19] reported that TIVA with short-acting SA in primary hip and knee arthroplasty provided good outcomes and earlier discharge. The current study showed that the type of anesthesia for geriatric hip fracture surgery influenced short-term outcomes; however, there was no significant difference in the long-term outcomes. Further prospective studies with large sample sizes and long-term follow-ups are necessary.

It has been recognized that minimizing opioid use and early rehabilitation improve clinical outcomes. FICB is a fast, minimal training-requiring local anesthesia technique for hip fracture surgery in recent years [16, 20–22]. Compared with SA, fewer complications and lesser discomfort associated with body positioning have been reported with FICB [17, 22–24]. Several randomized controlled trials, which evaluated the outcomes of FICB in patients undergoing hip fracture surgery, have demonstrated lower VAS scores with less morphine consumption and earlier postoperative ambulation. However, in this series, all patients received GA [16, 17, 25, 26]. Our study showed similar results. However, patients in our series received TIVA combined with FICB. With the advantages of TIVA, this novel anesthesia technique can optimize clinical outcomes.

Additional advantages of FICB + TIVA were found intraoperatively and postoperatively in our study. A significantly higher proportion of patients in Group II (11; 42%) had postoperative anemia and pneumonia than those in Group I. This might be because intraoperative blood pressure and vital signs can be easily controlled in patients receiving TIVA, which reduces blood loss during surgery [8, 15]. There was no intraoperative or postoperative neurologic complication or seizure attack in Group I. Additionally, more patients in Group II than in Group I (*p* = 0.03) had postoperative pneumonia caused by the insertion of an endotracheal tube. There were potential benefits such as avoidance of postoperative ventilator and critical care because of no intubation in Group I. This might have also led to lower ICU requirements and shorter length of ICU and hospital stay in Group I.

There are several limitations to our study. First, as a retrospective study, the anesthesia technique for patients under hip fracture surgery was based on anesthesiologists’ preference. Because the choice of anesthesia technique was not random, it might have led to a selective bias. Second, most of patients with ASA IV who suffered from hip fracture may choose non-operative treatment due to their poor conditions to receive an operation. With a small sample size, it would influence patients’ demographic data. Besides, we were unable to conclude that FICB + TIVA has definite benefits over GA for hip fracture surgery in high-risk geriatric patients. Third, the patients were analyzed by anesthesia type rather than by the injury type, condition, or procedure they underwent, which might also influence postoperative outcomes. Lastly, our conclusions cannot be extrapolated to elderly patients with ASA IV undergoing surgery for pathologic hip fracture or non-traumatic total hip replacement.

The strength of our study is that we evaluated elderly patients with high surgical risk (ASA grade IV) undergoing surgery for fragility hip fracture using the novel combined anesthesia technique: FICB + TIVA. Although a previous study showed the high mortality and clinical intractability in this population [4, 6], there is no literature focusing on the strategies to minimize intraoperative and postoperative complications and optimize clinical outcomes. Second, with the detailed intraoperative data, we could evaluate the intraoperative parameters and postoperative complications associated with patient outcomes in both groups.

A further prospective study with a large sample size to evaluate the long-term benefits of FICB + TIVA will be performed. We hope to establish a protocol for these high-surgical risk patients with fragility hip fractures. The new anesthesia technique might also be considered an alternative for other orthopedic injuries, such as distal femur fractures or procedures such as total joint replacement in patients with high surgical risk.

## Conclusion

For geriatric patients with ASA grade IV undergoing hip fracture surgery, FICB + TIVA showed several advantages over GA, such as lesser ICU requirement and shorter lengths of ICU stays. Moreover, less postoperative opioid use promoted earlier postoperative rehabilitation, shorter length of hospital stay, and optimized clinical outcomes. Although well-designed, randomized controlled trials with a large sample size are necessary to elucidate the long-term mortality, we recommend FICB + TIVA for geriatric patients undergoing hip fracture surgery, especially those with poor general health status and high surgical risk.

## Data Availability

There is no data obtained for this report.
